# (*E*)-4-[(4-Diethyl­amino-2-hy­droxy­benzyl­idene)amino]­benzonitrile

**DOI:** 10.1107/S1600536812008082

**Published:** 2012-02-29

**Authors:** Ming-Jen Chang, Tzu-Chien Fang, Hsing-Yang Tsai, Ming-Hui Luo, Kew-Yu Chen

**Affiliations:** a Department of Chemical Engineering, Feng Chia University, 40724 Taichung, Taiwan

## Abstract

The title compound, C_18_H_19_N_3_O, displays an *E* conformation with respect to the C=N double bond. The dihedral angle between the mean planes of the two benzene rings is 24.49 (3)°. An intra­molecular O—H⋯N hydrogen bond generates an *S*(6) ring. In the crystal, mol­ecules are linked by nonclassical inter­molecular C—H⋯O hydrogen bonds to form an infinite one-dimensional chain along [010], generating a *C*(8) motif.

## Related literature

For the preparation of the title compound, see: Shirinian *et al.* (2010 ▶). For the applications of proton transfer dyes, see: Chen & Pang (2010 ▶); Chuang *et al.* (2011 ▶); Han *et al.* (2010 ▶); Helal *et al.* (2010 ▶); Ikeda *et al.* (2010 ▶); Ito *et al.* (2011 ▶); Lim *et al.* (2011 ▶); Lins *et al.* (2010 ▶); Maupin *et al.* (2011 ▶); Santos *et al.* (2011 ▶); Tang *et al.* (2011 ▶). For related structures, see: Blagus & Kaitner (2011 ▶); Chen *et al.* (2011 ▶); Guo (2010 ▶); Manvizhi *et al.* (2011 ▶); Wang *et al.* (2010 ▶).
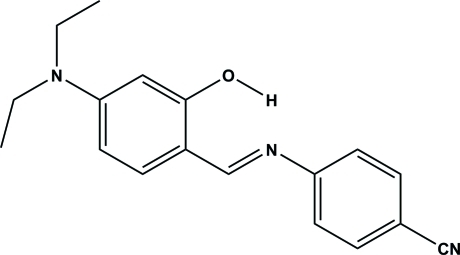


## Experimental

### 

#### Crystal data

C_18_H_19_N_3_O
*M* *_r_* = 293.36
Monoclinic, 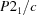

*a* = 15.361 (3) Å
*b* = 12.118 (2) Å
*c* = 8.7317 (14) Å
β = 100.717 (4)°
*V* = 1597.0 (5) Å^3^
*Z* = 4
Mo *K*α radiation
μ = 0.08 mm^−1^
*T* = 295 K
0.42 × 0.35 × 0.10 mm

#### Data collection

Bruker SMART CCD diffractometer
Absorption correction: multi-scan (*SADABS*; Bruker 2001 ▶) *T*_min_ = 0.436, *T*_max_ = 1.000
8867 measured reflections
3136 independent reflections
1405 reflections with *I* > 2σ(*I*)
*R*_int_ = 0.054

#### Refinement

*R*[*F* ^2^ > 2σ(*F* ^2^)] = 0.055
*wR*(*F* ^2^) = 0.171
*S* = 1.02
3136 reflections
191 parameters
H-atom parameters constrained
Δρ_max_ = 0.13 e Å^−3^
Δρ_min_ = −0.22 e Å^−3^

### 

Data collection: *SMART* (Bruker, 2001 ▶); cell refinement: *SAINT* (Bruker, 2001 ▶); data reduction: *SAINT*; program(s) used to solve structure: *SHELXS97* (Sheldrick, 2008 ▶); program(s) used to refine structure: *SHELXL97* (Sheldrick, 2008 ▶); molecular graphics: *ORTEP-3* (Farrugia, 1997 ▶); software used to prepare material for publication: *WinGX* (Farrugia, 1999 ▶).

## Supplementary Material

Crystal structure: contains datablock(s) I, global. DOI: 10.1107/S1600536812008082/rk2339sup1.cif

Structure factors: contains datablock(s) I. DOI: 10.1107/S1600536812008082/rk2339Isup2.hkl

Supplementary material file. DOI: 10.1107/S1600536812008082/rk2339Isup3.cml

Additional supplementary materials:  crystallographic information; 3D view; checkCIF report

## Figures and Tables

**Table 1:** Hydrogen-bond geometry (Å, °)

*D*—H⋯*A*	*D*—H	H⋯*A*	*D*⋯*A*	*D*—H⋯*A*
O—H0*A*⋯N2	0.82	1.84	2.572 (3)	148
C4—H4*A*⋯O^i^	0.93	2.60	3.334 (3)	137

Symmetry code: (i) 
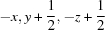
.

**Fig. 1.**
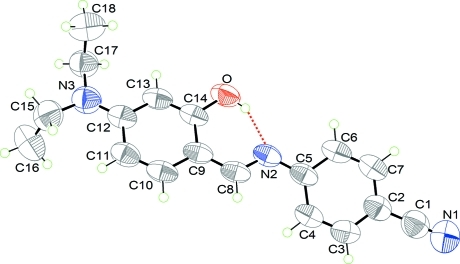
The molecular structure of the title compound, showing the atom-labelling scheme and the intramolecular O–H···N hydrogen bond (red dashed line). Displacement ellipsoids are drawn at the 50% probability level. H atoms are presented as a small spheres of arbitrary radius.

**Fig. 2.**
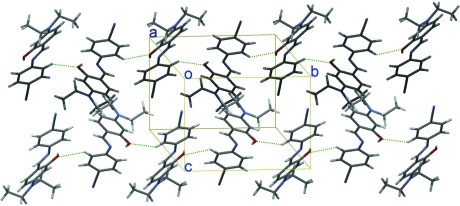
A section of the crystal packing of the title compound, viewed along the a axis. Green dashed lines denote the non-classical intermolecular C4–H4A···O hydrogen bonds.

## supplementary crystallographic information

### Comment

The excited-state intramolecular proton transfer (*ESIPT*) reaction of *N*-(2-hydroxybenzylidene)aniline derivatives has been investigated, which incorporates transfer of a hydroxy proton to the imine nitrogen through an intramolecular six-membered-ring hydrogen-bonding system. The proton transfer dyes have found many important applications. Prototypical examples are probes for solvation dynamics (Chen & Pang, 2010; Lins *et al.*, 2010) and biological environments (Lim *et al.*, 2011; Maupin *et al.*, 2011), photochromic materials (Ito *et al.*, 2011), near-infrared fluorescent dyes (Ikeda *et al.*, 2010), fluorescence microscopy imaging (Santos *et al.*, 2011), chemosensors (Han *et al.*, 2010; Helal *et al.*, 2010) and recent application in the field of organic light emitting devices (Chuang *et al.*, 2011; Tang *et al.*, 2011).

The molecular structure of the title compound is shown in Fig. 1. The molecule displays a *trans* configuration about the central C═N imine double bond (Blagus & Kaitner, 2011; Guo, 2010; Manvizhi *et al.*, 2011). The dihedral angle between the mean plane of two benzene rings is 24.49 (3)° (Wang *et al.*, 2010) and an intramolecular O–H···N hydrogen bond (Table 1) generates an S(6) ring (Chen *et al.*, 2011). In the crystal (Fig. 2), molecules are linked by non-classical intermolecular C–H···O hydrogen bonds (Table 1) to form an infinite one-dimensional chain along [0 1 0], generating a C(8) motif.

### Experimental

The title compound was synthesized by the condensation reaction of 4-(diethylamino)-2-hydroxybenzaldehyde and 4-aminobenzonitrile according to the literature (Shirinian *et al.*, 2010). Yellow parallelepiped crystals suitable for the crystallographic studies reported here were isolated over a period of five weeks by slow evaporation from a chloroform solution.

### Refinement

H atoms bonded to O and C atoms were located in a difference electron density map. The hydroxy H atom was freely refined, and other H atoms positioned geometrically and refined using a riding model, with C–H = 0.93Å-0.97Å and *U*_iso_(H) = 1.2(1.5)*U*_eq_(C)].

### Crystal data

**Table:**

C_18_H_19_N_3_O	*F*(000) = 624
*M**_r_* = 293.36	*D*_x_ = 1.220 Mg m^−^^3^
Monoclinic, *P*2_1_/*c*	Mo *K*α radiation, λ = 0.71073 Å
Hall symbol: -P 2ybc	Cell parameters from 1646 reflections
*a* = 15.361 (3) Å	θ = 2.2–22.6°
*b* = 12.118 (2) Å	µ = 0.08 mm^−^^1^
*c* = 8.7317 (14) Å	*T* = 295 K
β = 100.717 (4)°	Parallelepiped, yellow
*V* = 1597.0 (5) Å^3^	0.42 × 0.35 × 0.10 mm
*Z* = 4	

### Data collection

**Table:**

Bruker SMART CCD diffractometer	3136 independent reflections
Radiation source: fine-focus sealed tube	1405 reflections with *I* > 2σ(*I*)
Graphite monochromator	*R*_int_ = 0.054
φ and ω scans	θ_max_ = 26.1°, θ_min_ = 2.2°
Absorption correction: multi-scan (*SADABS*; Bruker 2001)	*h* = −18→18
*T*_min_ = 0.436, *T*_max_ = 1.000	*k* = −14→14
8867 measured reflections	*l* = −7→10

### Refinement

**Table:**

Refinement on *F*^2^	Primary atom site location: structure-invariant direct methods
Least-squares matrix: full	Secondary atom site location: difference Fourier map
*R*[*F*^2^ > 2σ(*F*^2^)] = 0.055	Hydrogen site location: inferred from neighbouring sites
*wR*(*F*^2^) = 0.171	H-atom parameters constrained
*S* = 1.02	*w* = 1/[σ^2^(*F*_o_^2^) + (0.075*P*)^2^] where *P* = (*F*_o_^2^ + 2*F*_c_^2^)/3
3136 reflections	(Δ/σ)_max_ < 0.001
191 parameters	Δρ_max_ = 0.13 e Å^−^^3^
0 restraints	Δρ_min_ = −0.22 e Å^−^^3^

### Special details

**Table:**

Geometry. All s.u.'s (except the s.u. in the dihedral angle between two l.s. planes) are estimated using the full covariance matrix. The cell s.u.'s are taken into account individually in the estimation of s.u.'s in distances, angles and torsion angles; correlations between s.u.'s in cell parameters are only used when they are defined by crystal symmetry. An approximate (isotropic) treatment of cell s.u.'s is used for estimating s.u.'s involving l.s. planes.
Refinement. Refinement of *F*^2^ against ALL reflections. The weighted *R*-factor *wR* and goodness of fit *S* are based on *F*^2^, conventional *R*-factors *R* are based on *F*, with *F* set to zero for negative *F*^2^. The threshold expression of *F*^2^ > σ(*F*^2^) is used only for calculating *R*-factors(gt) *etc*. and is not relevant to the choice of reflections for refinement. *R*-factors based on *F*^2^ are statistically about twice as large as those based on *F*, and *R*-factors based on ALL data will be even larger.

### Fractional atomic coordinates and isotropic or equivalent isotropic displacement parameters (Å^2^)

**Table:**

	*x*	*y*	*z*	*U*_iso_*/*U*_eq_	
O	−0.12526 (16)	−0.02917 (13)	0.1327 (2)	0.0914 (6)	
H0A	−0.0756	−0.0162	0.1827	0.137 (16)*	
N1	0.3699 (2)	0.1233 (3)	0.8181 (4)	0.1306 (11)	
N2	0.01778 (14)	0.08093 (15)	0.2272 (3)	0.0735 (6)	
N3	−0.34058 (17)	0.11544 (19)	−0.2774 (3)	0.0937 (6)	
C1	0.3116 (2)	0.1190 (2)	0.7158 (4)	0.0969 (9)	
C2	0.23844 (18)	0.1117 (2)	0.5869 (3)	0.0811 (7)	
C3	0.20738 (19)	0.2036 (2)	0.4999 (4)	0.0871 (8)	
H3A	0.2351	0.2714	0.5233	0.105*	
C4	0.13636 (18)	0.1961 (2)	0.3796 (3)	0.0810 (8)	
H4A	0.1161	0.2590	0.3230	0.097*	
C5	0.09401 (18)	0.09504 (19)	0.3410 (3)	0.0702 (7)	
C6	0.1265 (2)	0.0040 (2)	0.4290 (3)	0.0865 (8)	
H6A	0.0992	−0.0641	0.4061	0.104*	
C7	0.1975 (2)	0.0111 (2)	0.5485 (3)	0.0896 (8)	
H7A	0.2185	−0.0518	0.6043	0.107*	
C8	−0.00756 (15)	0.14955 (19)	0.1149 (3)	0.0701 (5)	
H8A	0.0292	0.2083	0.1014	0.084*	
C9	−0.08947 (16)	0.13804 (18)	0.0117 (3)	0.0701 (5)	
C10	−0.11940 (19)	0.21569 (19)	−0.1058 (3)	0.0776 (8)	
H10A	−0.0825	0.2744	−0.1187	0.093*	
C11	−0.1997 (2)	0.2089 (2)	−0.2014 (3)	0.0807 (8)	
H11A	−0.2159	0.2620	−0.2784	0.097*	
C12	−0.25904 (18)	0.1226 (2)	−0.1858 (3)	0.0749 (7)	
C13	−0.23028 (19)	0.04469 (19)	−0.0681 (3)	0.0778 (7)	
H13A	−0.2681	−0.0127	−0.0535	0.093*	
C14	−0.14852 (19)	0.05040 (18)	0.0257 (3)	0.0706 (7)	
C15	−0.3678 (2)	0.1867 (3)	−0.4140 (3)	0.1043 (10)	
H15A	−0.4051	0.1449	−0.4956	0.125*	
H15B	−0.3156	0.2101	−0.4532	0.125*	
C16	−0.4175 (2)	0.2868 (3)	−0.3755 (4)	0.1351 (13)	
H16A	−0.4337	0.3316	−0.4670	0.203*	
H16B	−0.3805	0.3287	−0.2954	0.203*	
H16C	−0.4700	0.2639	−0.3394	0.203*	
C17	−0.40588 (19)	0.0308 (2)	−0.2501 (3)	0.0937 (6)	
H17A	−0.4654	0.0586	−0.2864	0.112*	
H17B	−0.3990	0.0167	−0.1392	0.112*	
C18	−0.3941 (3)	−0.0743 (3)	−0.3325 (5)	0.1237 (12)	
H18A	−0.3371	−0.1052	−0.2905	0.164 (17)*	
H18B	−0.3979	−0.0598	−0.4417	0.20 (2)*	
H18C	−0.4396	−0.1255	−0.3185	0.187 (17)*	

### Atomic displacement parameters (Å^2^)

**Table:**

	*U*^11^	*U*^22^	*U*^33^	*U*^12^	*U*^13^	*U*^23^
O	0.1181 (17)	0.0591 (11)	0.0989 (14)	−0.0112 (10)	0.0253 (13)	0.0149 (10)
N1	0.110 (2)	0.132 (3)	0.143 (3)	0.0031 (19)	0.005 (2)	0.009 (2)
N2	0.0928 (16)	0.0518 (12)	0.0824 (15)	0.0057 (11)	0.0329 (13)	−0.0054 (11)
N3	0.1002 (14)	0.0906 (14)	0.0938 (13)	−0.0076 (10)	0.0270 (11)	0.0048 (11)
C1	0.089 (2)	0.091 (2)	0.115 (3)	0.0063 (19)	0.030 (2)	0.008 (2)
C2	0.0857 (19)	0.0719 (18)	0.093 (2)	0.0088 (16)	0.0367 (16)	0.0002 (16)
C3	0.097 (2)	0.0649 (17)	0.104 (2)	−0.0063 (15)	0.0315 (18)	−0.0012 (16)
C4	0.099 (2)	0.0546 (15)	0.094 (2)	0.0036 (14)	0.0300 (18)	0.0045 (14)
C5	0.0879 (18)	0.0504 (15)	0.0823 (18)	0.0087 (13)	0.0416 (15)	−0.0015 (13)
C6	0.113 (2)	0.0522 (16)	0.099 (2)	0.0024 (15)	0.0320 (19)	−0.0009 (15)
C7	0.114 (2)	0.0623 (18)	0.097 (2)	0.0123 (16)	0.031 (2)	0.0082 (15)
C8	0.0912 (13)	0.0506 (9)	0.0793 (14)	−0.0041 (10)	0.0439 (10)	−0.0043 (10)
C9	0.0912 (13)	0.0506 (9)	0.0793 (14)	−0.0041 (10)	0.0439 (10)	−0.0043 (10)
C10	0.107 (2)	0.0544 (15)	0.0822 (18)	−0.0098 (14)	0.0462 (17)	0.0021 (14)
C11	0.109 (2)	0.0652 (16)	0.0757 (18)	−0.0014 (16)	0.0364 (17)	0.0059 (13)
C12	0.0949 (19)	0.0610 (15)	0.0773 (18)	−0.0038 (15)	0.0380 (16)	−0.0025 (13)
C13	0.099 (2)	0.0571 (15)	0.0863 (19)	−0.0138 (14)	0.0393 (16)	−0.0013 (14)
C14	0.100 (2)	0.0471 (13)	0.0736 (17)	0.0011 (14)	0.0384 (16)	0.0006 (12)
C15	0.119 (2)	0.116 (3)	0.079 (2)	−0.010 (2)	0.0204 (18)	0.0069 (18)
C16	0.160 (3)	0.121 (3)	0.126 (3)	0.040 (3)	0.032 (2)	0.031 (2)
C17	0.1002 (14)	0.0906 (14)	0.0938 (13)	−0.0076 (10)	0.0270 (11)	0.0048 (11)
C18	0.134 (4)	0.110 (3)	0.135 (4)	−0.024 (3)	0.048 (3)	−0.024 (2)

### Geometric parameters (Å, º)

**Table:**

O—C14	1.344 (3)	C9—C10	1.405 (3)
O—H0A	0.8200	C9—C14	1.417 (3)
N1—C1	1.143 (4)	C10—C11	1.357 (3)
N2—C8	1.290 (3)	C10—H10A	0.9300
N2—C5	1.398 (3)	C11—C12	1.410 (3)
N3—C12	1.358 (3)	C11—H11A	0.9300
N3—C17	1.484 (3)	C12—C13	1.405 (3)
N3—C15	1.469 (3)	C13—C14	1.368 (3)
C1—C2	1.437 (4)	C13—H13A	0.9300
C2—C3	1.383 (3)	C15—C16	1.504 (4)
C2—C7	1.383 (3)	C15—H15A	0.9700
C3—C4	1.369 (3)	C15—H15B	0.9700
C3—H3A	0.9300	C16—H16A	0.9600
C4—C5	1.398 (3)	C16—H16B	0.9600
C4—H4A	0.9300	C16—H16C	0.9600
C5—C6	1.384 (3)	C17—C18	1.490 (4)
C6—C7	1.365 (3)	C17—H17A	0.9700
C6—H6A	0.9300	C17—H17B	0.9700
C7—H7A	0.9300	C18—H18A	0.9600
C8—C9	1.412 (3)	C18—H18B	0.9600
C8—H8A	0.9300	C18—H18C	0.9600
			
C14—O—H0A	109.5	C12—C11—H11A	119.5
C8—N2—C5	123.8 (2)	N3—C12—C13	121.2 (2)
C12—N3—C17	121.8 (2)	N3—C12—C11	122.2 (3)
C12—N3—C15	122.2 (2)	C13—C12—C11	116.6 (3)
C17—N3—C15	116.0 (2)	C14—C13—C12	122.2 (2)
N1—C1—C2	179.0 (4)	C14—C13—H13A	118.9
C3—C2—C7	118.8 (3)	C12—C13—H13A	118.9
C3—C2—C1	121.3 (3)	O—C14—C13	118.4 (2)
C7—C2—C1	119.9 (3)	O—C14—C9	120.4 (3)
C2—C3—C4	120.8 (3)	C13—C14—C9	121.2 (2)
C2—C3—H3A	119.6	N3—C15—C16	111.8 (2)
C4—C3—H3A	119.6	N3—C15—H15A	109.2
C3—C4—C5	120.8 (3)	C16—C15—H15A	109.2
C3—C4—H4A	119.6	N3—C15—H15B	109.2
C5—C4—H4A	119.6	C16—C15—H15B	109.2
N2—C5—C6	117.7 (2)	H15A—C15—H15B	107.9
N2—C5—C4	124.7 (2)	C15—C16—H16A	109.5
C6—C5—C4	117.5 (3)	C15—C16—H16B	109.5
C5—C6—C7	121.9 (3)	H16A—C16—H16B	109.5
C5—C6—H6A	119.1	C15—C16—H16C	109.5
C7—C6—H6A	119.1	H16A—C16—H16C	109.5
C2—C7—C6	120.3 (3)	H16B—C16—H16C	109.5
C2—C7—H7A	119.9	N3—C17—C18	111.5 (2)
C6—C7—H7A	119.9	N3—C17—H17A	109.3
N2—C8—C9	121.9 (2)	C18—C17—H17A	109.3
N2—C8—H8A	119.0	N3—C17—H17B	109.3
C9—C8—H8A	119.0	C18—C17—H17B	109.3
C10—C9—C14	115.9 (3)	H17A—C17—H17B	108.0
C10—C9—C8	122.1 (2)	C17—C18—H18A	109.5
C14—C9—C8	121.9 (2)	C17—C18—H18B	109.5
C11—C10—C9	123.0 (2)	H18A—C18—H18B	109.5
C11—C10—H10A	118.5	C17—C18—H18C	109.5
C9—C10—H10A	118.5	H18A—C18—H18C	109.5
C10—C11—C12	121.0 (2)	H18B—C18—H18C	109.5
C10—C11—H11A	119.5		
			
C7—C2—C3—C4	1.2 (4)	C17—N3—C12—C13	4.9 (4)
C1—C2—C3—C4	−178.5 (2)	C15—N3—C12—C13	−171.7 (2)
C2—C3—C4—C5	−0.6 (4)	C17—N3—C12—C11	−173.9 (2)
C8—N2—C5—C6	−163.3 (2)	C15—N3—C12—C11	9.5 (4)
C8—N2—C5—C4	21.3 (3)	C10—C11—C12—N3	178.3 (2)
C3—C4—C5—N2	175.6 (2)	C10—C11—C12—C13	−0.5 (3)
C3—C4—C5—C6	0.2 (4)	N3—C12—C13—C14	−179.8 (2)
N2—C5—C6—C7	−176.2 (2)	C11—C12—C13—C14	−1.0 (3)
C4—C5—C6—C7	−0.5 (4)	C12—C13—C14—O	−178.2 (2)
C3—C2—C7—C6	−1.5 (4)	C12—C13—C14—C9	2.1 (4)
C1—C2—C7—C6	178.2 (2)	C10—C9—C14—O	178.8 (2)
C5—C6—C7—C2	1.1 (4)	C8—C9—C14—O	−4.4 (3)
C5—N2—C8—C9	−173.49 (19)	C10—C9—C14—C13	−1.5 (3)
N2—C8—C9—C10	176.6 (2)	C8—C9—C14—C13	175.3 (2)
N2—C8—C9—C14	0.0 (3)	C12—N3—C15—C16	−95.1 (3)
C14—C9—C10—C11	0.0 (3)	C17—N3—C15—C16	88.1 (3)
C8—C9—C10—C11	−176.8 (2)	C12—N3—C17—C18	−87.4 (3)
C9—C10—C11—C12	1.0 (4)	C15—N3—C17—C18	89.4 (3)

### Hydrogen-bond geometry (Å, º)

**Table:**

*D*—H···*A*	*D*—H	H···*A*	*D*···*A*	*D*—H···*A*
O—H0*A*···N2	0.82	1.84	2.572 (3)	148
C4—H4*A*···O^i^	0.93	2.60	3.334 (3)	137

Symmetry code: (i) −*x*, *y*+1/2, −*z*+1/2.
